# Marker assisted pedigree breeding based improvement of the Indian mega variety of rice MTU1010 for resistance against bacterial blight and blast and tolerance to low soil phosphorus

**DOI:** 10.1371/journal.pone.0260535

**Published:** 2022-01-31

**Authors:** Laxmi Prasanna B., Dangi K. S., Damodar Raju C. H., Jagadeeshwar R., Rekha G., Pragya Sinha, Aleena D., Harika G., Mastanbee S. K., Swapnil Ravindra K., Kousik M. B. V. N., Mahadeva Swamy H. K., Anila M., Kale R. R. R. R., Dilip Kumar T., Punniakotti E., Hajira S. K., Bhadana V. P., Sundaram R. M.

**Affiliations:** 1 Regional Agricultural Research Station, PJTSAU, Jagtial, India; 2 Professor Jayashankar Telangana State Agricultural University, Hyderabad, India; 3 Indian Institute of Rice Research, Hyderabad, India; 4 ICAR-Sugarcane Breeding Institute, Coimbatore, Tamil Nadu, India; 5 ICAR-Indian Institute of Agricultural Biotechnology (IIAB), Ranchi, India; National Institute of Plant Genome Research, INDIA

## Abstract

Rice production is affected by many biotic and abiotic stresses; among them, bacterial blight (BB) and blast diseases and low soil phosphorous stress cause significant yield losses. The present study was carried out with the objective of combining the BB resistance gene, *Xa21*, the blast resistance gene, *Pi54*, and the low soil phosphorous tolerance QTL/gene, *Pup1*, into the genetic background of the Indian mega-rice variety, MTU1010 (Cottondora Sannalu), through marker-assisted pedigree breeding. RP5973-20-9-8-24-12-7 [a near isogenic line (NIL) of MTU1010 possessing *Pup1*] and RP6132 [a NIL of Akshayadhan possessing *Xa21* + *Pi54*] were crossed and ‘true’ F_1_s were identified, using the target gene-specific markers and selfed. F_2_ plants, which are homozygous for all the three target genes/QTLs, were identified using PCR based markers and were advanced further through the pedigree method of breeding, with selection based on phenotypic traits specific for MTU1010. At the F_5_ generation, a set of 15 promising triple positive homozygous lines were identified and screened for their resistance against BB and blast diseases and tolerance to low soil P. Among them, two lines (LPK 30-18-16 and LPK 49-15-22) showed higher yields as compared to MTU1010, along with the desirable long slender grain type in both low soil P and normal soil P plots, and also exhibited high levels of resistance against BB and blast diseases, with lesser grain shattering as compared to MTU1010. These lines are being advanced for multi-location trials for validating their performance.

## Introduction

Rice is the predominant food crop of India in terms of area, production and consumption. In fact, the demand for rice is steadily increasing, as the number of rice consumers increase, especially in developing countries such as India [1]. It is predicted that the world population will exceed eight billion people in the next decade. Even though significant improvements have been witnessed in rice production following the advent of the Green Revolution, there is an imminent need to improve the productivity of rice (which has stagnated in the last decade or two), in order to meet the demands of an ever-growing population. This is indeed impeded by major biotic and abiotic stresses, causing huge yield losses in different rice growing ecosystems. Among the biotic stresses, two most devastating diseases in rice, namely blast, which is caused by the fungus *Magnaporthe oryzae*, and bacterial blight (BB), caused by *Xanthomonas oryzae pv*. *oryzae*, have plagued rice farmers since the beginning of rice cultivation throughout Asia and India [2]. BB and blast diseases can cause yield losses up to 74 to 81% [3] and 70 to 80% [1], respectively.

Among the abiotic stresses, low soil P stress is one of the major constraints on plant growth and yield worldwide, in many crops including rice [4, 5]. It is estimated that in more than 40% of arable lands, crop productivity is limited by P deficiency, and hence farmers are applying more and more phosphatic fertilizers, which in turn increase the cost of cultivation and pollution of water bodies. Genetic improvement through the deployment of resistant/tolerant genes is the most effective approach to prevent damage due to the two major biotic stresses, viz., BB, blast [6] and also low soil P levels [7].

MTU1010 (IET15644; also known as Cottondora Sannalu) is a medium-duration Indian mega-rice variety, released by the Andhra Pradesh Rice Research Station (APRRS), Acharya N.G. Ranga Agricultural University (ANGRAU), Maruteru, Andhra Pradesh, India, during the year 2000. It is a high-yielding rice variety, adaptable to multiple rice growing ecosystems, and has a desirable medium-slender grain type. However, despite these desirable features, MTU1010 is highly susceptible to BB and has only a moderate level of tolerance to blast disease, both of which cause significant yield losses in the variety. Furthermore, MTU1010 is also highly sensitive to low soil P levels, which reduces its production in problem soils. In order to cope with these three problems, development of resistant/tolerant breeding lines and their cultivation is considered the most effective, economical and environment friendly strategy. Considering the imminent need to improve BB and blast resistance along with low P tolerance, through this study, we attempted to deploy the strategy of marker-assisted pedigree breeding (MAPB) to transfer *Xa21*, *Pi54* and *Pup1* into the genetic back ground of MTU1010.

## Materials and methods

### Plant materials

A near-isogenic line (NIL) of MTU1010, viz., RP5973-20-9-8-24-12-7 possessing *Pup1* QTL, developed by the ICAR-Indian Institute of Rice Research (ICAR-IIRR) [7] was used as the female parent, while a NIL of Akshayadhan (RP6132) possessing BB (*Xa21*) and blast resistance (*Pi54*) genes, developed at ICAR-IIRR [8], was used as the male parent. Both RP5973-20-9-8-24-12-7 and RP6132 are of long slender grain type and belong to a medium-duration maturity group, akin to MTU1010. Improved Samba Mahsuri (ISM) and Swarna were used as sensitive and tolerant checks, respectively, under low soil P conditions, while TN1 and ISM were used as susceptible and resistant checks, respectively, in screening for BB resistance, and Tetep and HR12 were used as resistant and susceptible checks, respectively, in screening for blast resistance.

### Strategy for marker-assisted pedigree breeding

**Fig 1:**
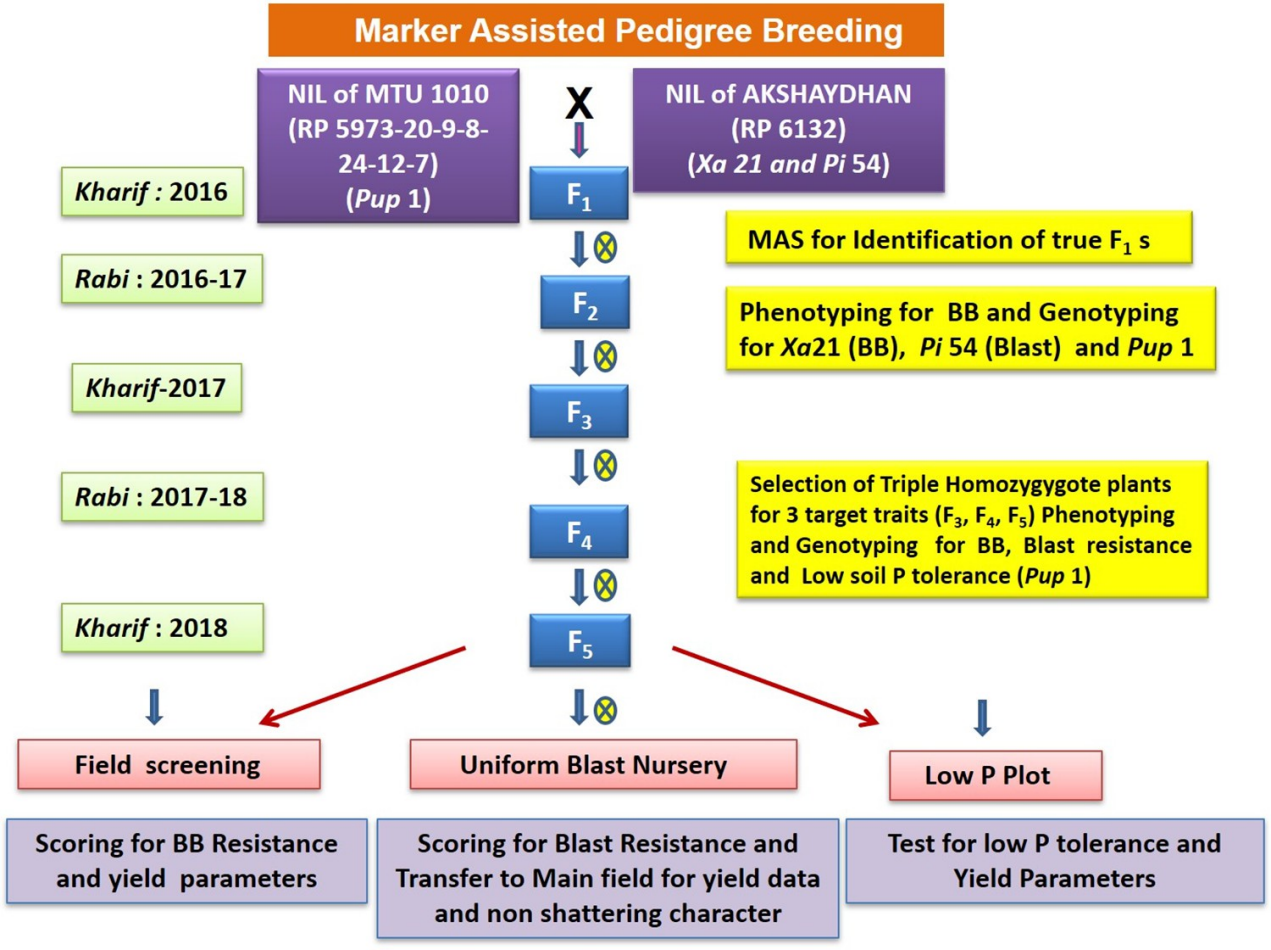
Schematic representation of marker assisted pedigree breeding.

A cross was carried out between RP 5973-20-9-8-24-12-7 and RP 6132 to combine their traits, low soil phosphorous tolerance (conferred by the QTL *Pup1*), BB resistance (conferred by the gene *Xa21*) and blast resistance (conferred by the gene *Pi54*) into the genetic background of MTU1010, during wet season (i.e., kharif season, 2016). The F_1_s were confirmed for their heterozygosity using target-resistant gene-specific markers, K-20-2_*Bspe1*_ (specific for *Pup1*) [9], Pi54MAS (specific for *Pi54*) [10] and pTA248 (specific for *Xa21*) [11], and the true F_1_ plants (i.e., heterozygous F_1_ plants) were selfed to develop F_2_s (Fig 1).

Homozygous positive F_2_ plants were identified through foreground selection, using the target QTLs/gene-specific markers mentioned above, and advanced further by selfing through pedigree breeding to F_5_ generation, based on selection for phenotypic characters specific for MTU1010. Marker-assisted selection was carried out through mini-scale rapid DNA isolation protocol, described in [12]. PCR protocols described in [13] were adopted for indirect selection of *Xa21*and *Pi54*, while the PCR protocol described in [7] was adopted for indirect selection of *Pup1*, using the markers mentioned earlier. Fifteen promising homozygous positive F_5_ lines, which closely resembled MTU1010 in terms of plant type and grain type, were selected and further evaluated for their resistance/tolerance to target stresses (i.e., BB and blast diseases and low soil phosphorous tolerance) as well as for key agro-morphological traits.

### Evaluation of BB, blast resistance, and low soil phosphorous tolerance in the improved breeding lines phenotypic screening for BB resistance

Screening for BB resistance was carried out among the selected F_5_ lines, along with the parents and check lines in the field at ICAR-IIRR, Hyderabad, during wet season (i.e., kharif season, 2018). The bacterial cultures of a virulent isolate of *Xanthomonas oryzae pv*. *Oryzae* (collected from Hyderabad, Telangana State, India), viz., DX-020 were maintained as per the protocols described by [14], and inoculation was done at maximum tillering stage by following the leaf clipping method described in [15]. The lesion length on the leaves were measured after 15 days of inoculation, and scoring was done as per the standard evaluation system (SES) of the International Rice Research Institute (IRRI), Philippines (IRRI, 2013).

### Phenotypic screening for blast resistance

The selected F_5_ lines along with the parents and checks-MTU1010 NIL (RP5973-20-9-8-24-12-7), Akshayadhan NIL (RP6132), susceptible check (HR12) and resistance check (Tetep) -were screened for blast resistance in the uniform blast nursery (UBN) at ICAR-IIRR, during wet season 2018. A local virulent isolated of the blast pathogen, *Magnaporthe grisea*, SPI-40, originally collected from Hyderabad, Telangana, India, was cultured and stored, as described by [16]. The young seedlings at the four-leaf stage were inoculated with the blast spores, as described in [13], and scored 15 days after inoculation, based on a 0 to 9 scale, as per IRRI-SES (IRRI, 2013).

### Phenotypic screening for low soil P tolerance

During wet season 2018, the selected F_5_ lines along with the parents and checks-MTU1010 NIL (RP5973-20-9-8-24-12-7), Akshayadhan NIL (RP6132), susceptible check (ISM) and resistance check (Swarna) were transplanted in the low soil P plot of ICAR-IIRR in a randomized block design of spacing 15cm x 20cm, in three replications. Except for the application of phosphorus, all the other agronomic practices including recommended doses of nitrogen, potassium and micro nutrients (i.e., Iron and zinc) were followed.

The following observations were recorded viz., days to 50% flowering (DFF), plant height (PH), number of productive tillers per plant (NPT), panicle length (PL), number of grains per panicle (GPP), dry shoot weight (DSW), dry root weight (DRW), root to shoot ratio (RSR), root volume (RV) and grain yield per plant (GY).

### Evaluation of agro-morphological traits in the improved breeding lines of MTU1010

During wet season 2018, the selected F_5_ lines possessing *Pup1*, *Xa21* and *Pi54*, and the parents, MTU1010 NIL (RP 5973-20-9-8-24-12-7) and Akshayadhan NIL (RP 6132), were transplanted into the experimental farm of ICAR-IIRR, in 2m^2^ plots in a randomized block design of spacing 15cmx20 cm in soil with optimum levels of macro and micro-nutrients. Data was recorded for the selected agro-morphological traits, i.e., days to 50% flowering (DFF), plant height (PH), number of productive tillers per plant (NPT), panicle length (PL), number of grains per panicle (GPP,nos.), dry shoot weight (DSW), dry root weight (DRW), root to shoot ratio (RSR), grain yield per plant (GY) and root volume (RV).

### Statistical analysis

Data was recorded for all the parameters in both normal soil P and low soil P plots, and statistical analysis was conducted using the SAS 9.2 (SAS version 9.2 software packages; SAS Institute, Inc.; Cary, NC) software for analysis of coefficient of variation (CV), critical difference (CD), standard error (SE) and analysis of variance (ANOVA).

## Results

### Combining BB, blast, and low P resistance/tolerance genes/QTLs into the genetic background of MTU 1010

**Fig 2:**
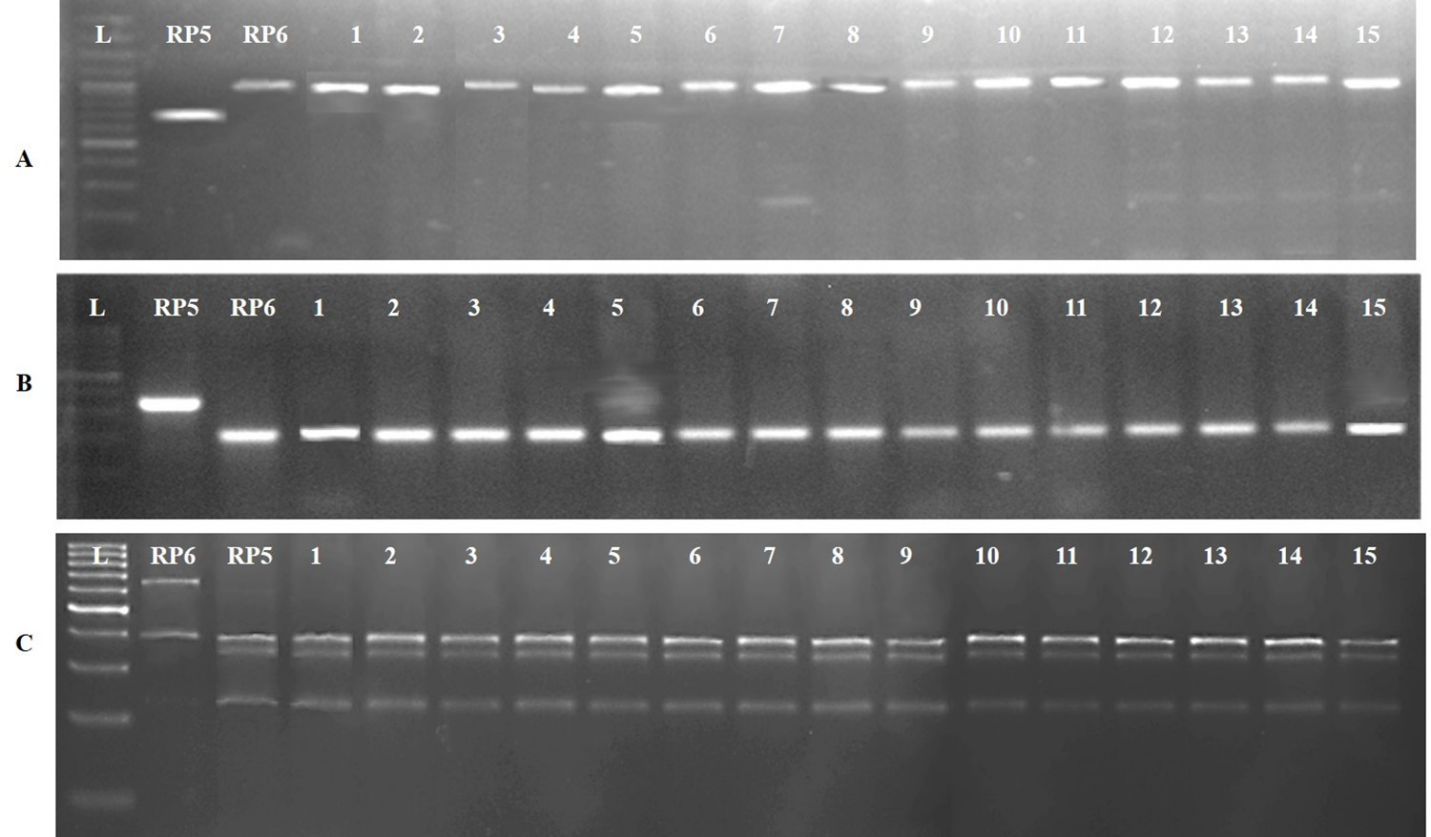
Screening of selected F_5_ generation lines with marker specific for target genes/QTL (A) Screening with pTA248 marker for *Xa21*, (B) Screening with Pi54MAS marker for *Pi54* (C) Screening with K20-2 *Bsp1* marker for *Pup1*. L-100bp ladder; RP: RP5973-20-9-8-24-12-7; *Pup1*, i.e., negative control for *Xa21* and *Pi54* and positive control for *Pup1*; RP: RP6132; Xa21 & *Pi54*, i.e., positive control for *Xa21* and *Pi54* and negative control for *Pup1*; 1–15: selected F_5_ lines. All the selected lines were homozygous for the target genes/QTL.

A total of 96 F_1_ seeds were generated from the cross between MTU1010 (RP5973-20-9-8-24-12-7, i.e., the female parent) and a NIL of Akshayadhan (RP6132, i.e., the male parent). Foreground selection was carried out with the target gene-specific markers, viz. K20-2, pTA248 and Pi54MAS, which are specific for *Pup1*, *Xa21* and *Pi54*, respectively. A total of 87 true F_1_s (heterozygous plants) were identified. They were then selfed to generate the F_2_ population consisting of 1267 plants. Phenotypic screening for BB resistance revealed a total of 980 resistant plants, which were further genotyped with *Xa21* gene-specific marker, pTA248, among which 315 plants were identified to be homozygous for the gene. They were then subjected to a screening using *Pi54* as the specific marker Pi54-MAS, and the *Pup1* specific co-dominant CAPS marker, K20-2. Among the 315 plants, 15 were found to be triple positive (i.e., triple homozygous for all the three target genes, i.e., *Pup1Pup1*, *Xa21Xa21*, *Pi54Pi54*) (Fig 2). These 15 plants were the advanced through pedigree breeding till F_5_ generation. In each generation of selfing, phenotypic selection was deployed for identification of promising plants, resembling MTU1010, based on agro-morphological traits.

### Assessment of the improved breeding lines under BB, blast and low P stresses

Phenotypic screening was carried out for fifteen homozygous F_5_ lines against three target stresses viz., low soil P, BB and blast diseases during wet season, 2018, at ICAR-IIRR, Hyderabad.

### Evaluation of improved breeding lines for bacterial leaf blight resistance

**Fig 3:**
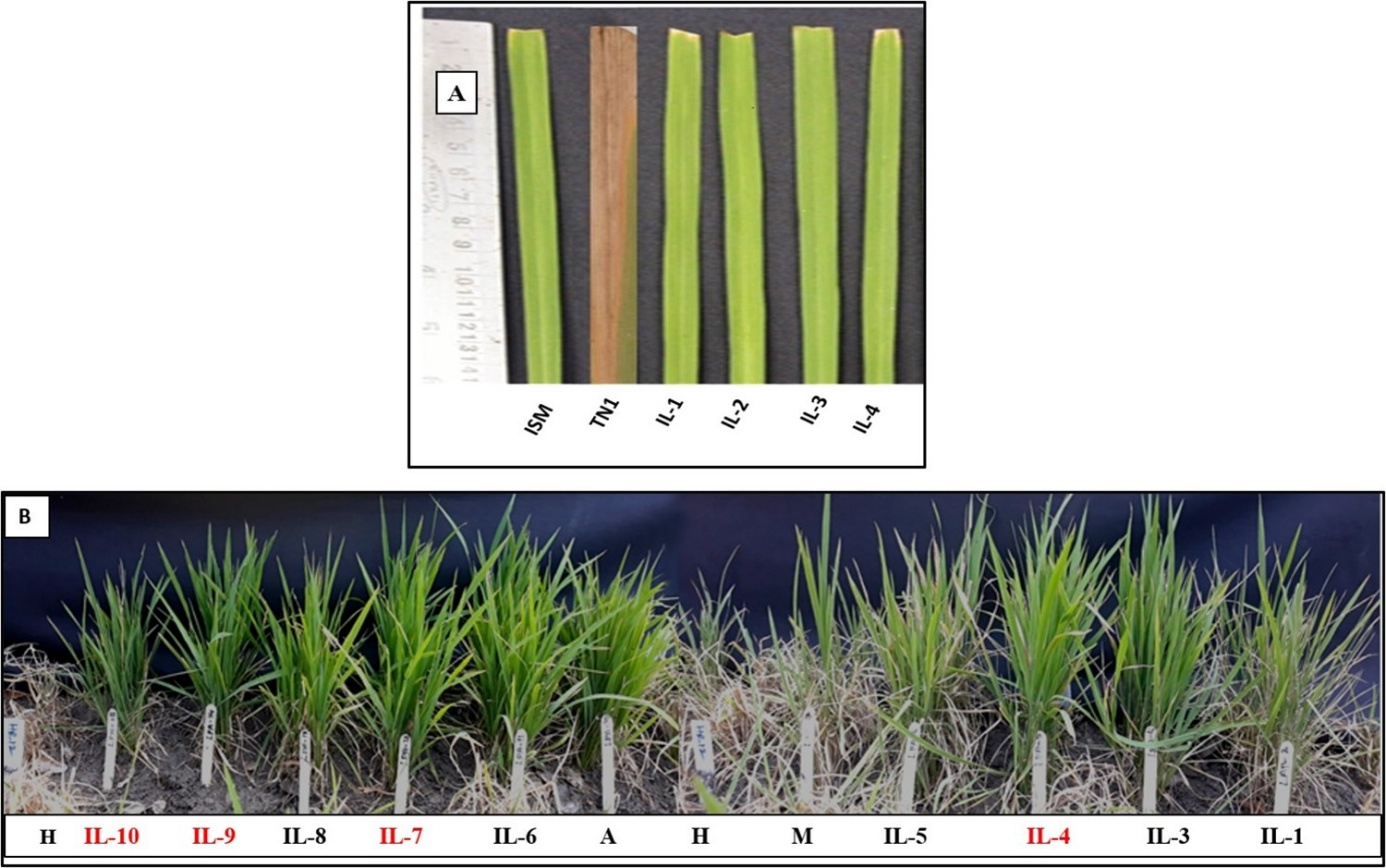
Screening of selected breeding lines at F_5_ generation for their resistance against bacterial blight and blast **(A)** Screening for bacterial blight resistance using *Xoo* (DX-020) culture. ISM- Resistant check; TN1- Susceptible check; IL-1 to IL-4- Improved breeding lines (LPK 28-19-15, LPK 38-18-16, LPK 49-1-21 and LPK 49-15-22) possessing *Pup1*, *Xa21 and Pi54* (F_5_ generation). **(B)** Phenotypic screening for blast resistance using SPI-40 fungal spore culture. A: Male Parent- Akshayadhan NIL (RP6132, (*Xa21 & Pi54*) H: Susceptible check-HR 12, M: Female Parent -MTU 1010 (RP5973-20-9-8-24-12-7; *Pup1*), IL-1 to IL-10: Improved breeding lines possessing *Pup1*, *Xa21* and *Pi54* (F_5_ Generation). All the selected breeding lines were resistant to bacterial blight and blast diseases. Four breeding lines i.e., IL-4: LPK 28-19-15, IL-7: LPK 38-18-16, IL-9: LPK 49-1-21 and IL-10: LPK 49-15-22, were highly resistant to blast disease with score 3).

The female parent RP5973-20-9-8-24-12-7 (NIL of MTU1010 with *Pup1*; susceptible to BB) and TN1 (susceptible check) were found to be highly susceptible to BB, with a mean lesion length of 15±0.3 (score 9), while the male parent (RP6132; BB resistant) and ISM (resistant check) were observed to be BB resistant, with a mean lesion length of 1±0.3 (score1). All the 15 breeding lines were observed to be resistant against BB, with mean lesion lengths ranging from 1± 0.3 to 3 ± 0.3. Among them, four lines-LPK28-19-15, LPK38-5-18, LPK49-1-21 and LPK49-21-20 exhibited excellent resistance with mean lesion lengths of 1±0.3 (score 1), similar to that of the resistant parent (Fig 3A; Table 1).

**Table 1:** Phenotypic screening of selected F_5_ breeding lines for BB and blast resistance at F_5_ generation.

Parents and Checks	Reaction against BB^$^			Reaction against Blast^#^	
	DX-020			SPI-40	
	**Lesion length (cm)**	**Score**	**I/R/MR/MS/S/HS**	**Score**	**R/MR/S**
RP5973-20-9-8-24-12-7	12± 0.3	9	S	9	S
RP6132	1± 0.3	1	R	3	R
ISM (Resistant check)	0± 0.0	1	R	1	R
TN1 (Susceptible check)	15± 0.3	9	S	9	S
**Improved breeding lines (F**_**3**_ **)**	**Lesion length (cm)**	**Score**	**R/MR/S**	**Score**	**R/MR/S**
LPK 3-19-3	3± 0.3	1	R	4	R
LPK 4-20-4	3± 0.3	1	R	4	R
LPK 10-1-5	3± 0.3	1	R	4	R
LPK 28-19-15	1± 0.3	1	R	3	R
LPK 28-13-16	3± 0.3	1	R	4	R
LPK 38-5-18	1± 0.3	1	R	3	R
LPK 30-18-16	3± 0.3	1	R	3	R
LPK 45-17-19	3± 0.3	1	R	4	R
LPK 49-1-21	1± 0.3	1	R	3	R
LPK 49-15-22	3± 0.3	1	R	3	R
LPK 49-21-20	1± 0.3	1	R	3	R
LPK 50-14-23	3± 0.3	1	R	4	R
LPK 54-11-24	3± 0.3	1	R	4	R
LPK 50-20-25	3± 0.3	1	R	4	R
LPK 42-18-26	3± 0.3	1	R	4	R

^$^The improved breeding lines at F_5_ generation (possessing *Pup1*, *Xa21*, and *Pi54*) were screened with a local virulent isolate of the bacterial blight pathogen, DX-020 under artificial conditions. R-Resistant, S-Susceptible.

^**#**^The improved breeding lines at F_5_ generation (possessing *Pup1*, *Xa21*, *Pi54*) were screened with a local virulent isolate of the blast pathogen, SPI-40 under controlled conditions in Uniform Blast Nursery (UBN). R-Resistant, S-Susceptible.

### Evaluation of improved breeding lines for blast resistance

The susceptible check TN1 and the female parent RP5973-20-9-8-24-12-7 were found to be highly susceptible to blast (score9), while the male parent RP6132 (score3) and Tetep (resistance check) (score1) were highly resistant to the disease. Among the 15 F_5_ lines screened for blast resistance, four breeding lines-LPK 28-19-15, LPK 38-5-18, LPK 49-1-21 and LPK 49-21-20 were found to be highly resistant to blast disease (score 3), similar to the resistant parent, and 11 lines showed resistance to the disease (score 4) (Fig 3B; Table 1).

### Evaluation of improved breeding lines for low soil P tolerance

Statistical analysis using ANOVA revealed significant difference between tolerant and sensitive parents, as well as the breeding lines for all the traits, except for DSW (Table 2). Under low soil P condition, the DFF of RP5973-20-9-8-24-12-7 (i.e., NIL of MTU1010 possessing *Pup1*) and RP6132 (possessing *Xa21*and *Pi54*), were 110 and 133 days, respectively.

**Table 2:** Evaluation of the improved breeding lines (F_5_ generation) in plot with low soil P at ICAR-IIRR during wet season 2018.

Genotype/ Entry	DFF	PH	NPT	PL	RV	RL	DSW	DRW	RSR	NGP	TGW	SPY	GT
RP5973-20-9-8-24-12-7	110.3 ± 0.9	78.7± 0.3	7.0 ± 0.6	18.0 ± 0.1	46.3 ± 0.3	23 ± 0.6	43.2 ± 0.4	10.5 ± 0.8	0.41± 0.0	112.0 ± 0.3	13.2 ± 0.4	8.9 ± 0.2	LS
RP (6132)	133.0 ± 0.6	66.0 ± 0.6	4.0 ± 0.3	11.9 ± 0.9	28.7 ± 0.3	33.3 ± 0.3	31.6 ± 0.9	3.7 ± 0.6	0.09± 0.0	87.0 ± 0.7	11.1 ± 0.2	4.2 ± 0.4	LS
ISM	127.0 ± 0.7	41.3 ± 0.6	2.3 ± 0.7	11.3 ± 0.9	12.6 ± 0.3	21.6 ± 0.3	24.5 ± 0.4	1.0 ± 0.3	0.08± 0.0	75 ± 0.3	11.5± 0.4	2.6 ± 0.2	MS
SWARNA	130.0 ± 0.3	63.6 ± 0.6	8.9 ± 0.3	18.7 ± 0.4	28.9 ± 0.3	30.5 ± 0.3	28.9 ± 0.3	2.4 ± 0.1	0.13± 0.0	115± 0.3	17.9± 0.3	9.8 ± 0.1	SB
LPK 3-19-3	113.0 ± 0.6	78.0 ± 0.6	7.0 ± 0.3	16.4 ± 0.8	36 ± 0.6	31.7 ± 0.9	41.9 ± 0.4	9.2 ± 0.8	0.30± 0.0	81.0 ± 0.9	10.4 ± 0.4	6.2 ± 0.2	LS
LPK 4-20-4	121.0 ± 0.6	78.0 ± 0.6	9.0 ± 0.3	19.2 ± 0.2	50.7 ± 0.7	32.0 ± 0.6	41.0 ± 0.6	6.7 ± 0.9	0.28± 0.0	110 ± 0.3	13.6 ± 0.4	8.8 ± 0.1	LS
LPK 10-1-5	124.0 ± 0.6	80.0 ± 0.6	6.0 ± 0.6	17.8 ± 0.3	35.7 ± 0.7	31.3 ± 0.3	36.0 ± 0.6	9.2 ± 0.5	0.30± 0.0	92.0 ± 0.9	10.1 ± 0.4	5.8 ± 0.1	LS
LPK 28-19-15	115.3 ± 0.3	81.0 ± 0.6	7.0 ± 0.3	14.1 ± 0.7	36.0 ± 0.6	27.7 ± 0.7	48.4 ± 0.8	7.2 ± 1.3	0.24± 0.0	78.0 ± 0.6	10.5 ± 0.5	4.4 ± 0.2	LS
LPK 28-13-16	121.0 ± 0.6	79.3 ± 0.3	5.0 ± 0.3	16.4 ± 0.6	34.3 ± 0.3	26.0 ± 0.6	49.0 ± 0.3	7.3 ± 0.3	0.23± 0.0	81 ± 0.9	10.1 ± 0.5	7.7 ± 0.3	LS
LPK 30-18-16	108.7 ± 0.3	80.3 ± 0.9	8.0 ± 0.3	19.5 ± 0.3	46.7 ± 0.3	29.5 ± 0.8	38.4 ± 0.5	8.5 ± 0.8	0.20± 0.0	121 ± 0.9	13.0 ± 0.5	9.2 ± 0.5	LS
LPK 38-5-18	124.3 ± 0.3	77.0 ± 0.6	6.0 ± 0.3	15.5 ± 0.4	41.7 ± 0.9	23.7 ± 0.7	31.3 ± 0.2	8.9 ± 0.5	0.28± 0.0	70.0 ± 0.6	11.4 ± 0.7	6.9 ± 0.1	LS
LPK 45-17-19	126.7 ± 0.7	79.7 ± 0.9	5.0 ± 0.3	15.0 ± 0.7	42.7 ± 0.9	27.3 ± 0.7	49.3 ± 0.9	7.6 ± 0.3	0.31± 0.0	83.0 ± 0.7	12.1 ± 0.2	5.8 ± 0.2	LS
LPK 49-21-20	133.0 ± 0.6	80.7 ± 0.3	4.0 ± 0.6	13.3 ± 0.3	46.0 ± 0.6	26.2 ± 0.4	36.2 ± 0.4	9.0 ± 0.6	0.23± 0.0	73.0 ± 0.6	11.0 ± 0.7	8.3 ± 0.3	LS
LPK 49-1-21	121.3 ± 0.3	81.0 ± 0.6	9.0 ± 0.3	18.0 ± 0.3	52.0 ± 0.6	28.6 ± 0.7	47.0 ± 0.6	4.4 ± 0.2	0.30± 0.0	113.0 ± 0.7	17.1 ± 0.3	9.9 ± 0.6	LS
LPK 49-15-22	111.3 ± 0.3	80.7 ± 0.3	8.0 ± 0.3	18.2 ± 0.2	49.7 ± 0.9	29.3 ± 0.3	32.9 ± 0.5	6.3 ± 0.8	0.25± 0.0	121.0 ± 0.6	17.0 ± 0.3	9.9 ± 0.2	LS
LPK 50-14-23	128.7 ± 0.3	78.3 ± 0.3	7.0 ± 0.3	14.6 ± 0.9	43.0 ± 0.6	23.3 ± 0.9	30.7 ± 0.3	9.3 ± 0.8	0.45± 0.0	89.0 ± 0.3	10.0 ± 0.5	5.7 ± 0.3	LS
LPK 54-11-24	122.7 ± 0.6	78.7 ± 0.3	6.0 ± 0.6	16.9 ± 0.3	44.3 ± 0.9	33.0 ± 0.6	31.3 ± 0.3	5.7 ± 0.3	0.27± 0.0	73.0 ± 0.7	12.3 ± 0.7	4.8 ± 0.2	LS
LPK 50-20-25	120.7 ± 0.3	73.0 ± 0.6	4.0 ± 0.6	14.0 ± 0.8	43.6 ± 0.3	27.7 ± 0.7	47.7 ± 0.3	9.9 ± 0.5	0.26± 0.0	91.0 ± 0.6	10.9 ± 0.6	6.0 ± 0.3	LS
LPK 42-18-26	128.3 ± 0.7	81.7 ± 0.3	7.0 ± 0.6	14.3 ± 0.4	46.3 ± 0.6	31.2 ± 0.6	31.7 ± 0.3	8.8 ± 0.8	0.38± 0.0	79.0 ± 0.3	10.5 ± 0.8	6.3 ± 0.3	LS
**CV%**	0.77	1.25	11.61	5.86	2.50	3.80	2.38	14.20	19.60	1.13	7.23	6.73	
**LSD at 5%**	1.55	1.57	1.22	1.54	1.66	1.74	1.49	1.67	0.08	1.79	1.42	0.76	
**F value**	184.60	283.99	14.59	18.12	275.69	35.77	233.03	21.38	9.93	821.10	12.66	62.06	
**p value (1%)**	< .0001	< .0001	< .0001	< .0001	< .0001	< .0001	< .0001	< .0001	< .0001	< .0001	< .0001	< .0001	

RP5973-20-9-8-24-12-7 Female parent; RP(6132)- Male Parent; DFF-days to 50% flowering, PH-plant height (cm), NPT-number of productive tillers per plant, PL-panicle length (cm), RV-root volume (ml), DSW-dry shoot weight (gr), DRW-dry root weight (gr), RSR-root to shoot ratio (%), NGP-grain yield/plant, TGW-1000 grain weight, SPY-single plant yield, SB-Short Bold, LS-Long slender, MS-Medium slender; CV-Coefficient variance; CD-Critical differential at 5% probability level. ±Standard error and values given are mean of three replication.

With respect to the improved breeding lines (i.e., F_5_ generation lines possessing *Pup1*, *Xa21*, and *Pi54*), DFF varied from 108 days (LPK 30-18-16) to 133 days (LPK 49-21-20). One breeding line, LPK 30-18-16 (108 days), flowered significantly earlier as compared to the female parent, RP5973-20-9-8-24-12-7 (MTU1010 NIL). The breeding lines LPK 3-19-3, LPK 4-20-4, LPK 10-1-5, LPK 28-19-15, LPK 28-13-16, LPK 38-5-18, LPK 45-17-19, LPK 49-21-20, LPK 49-1-21, LPK 49-15-22, LPK 50-14-23, LPK 54-11-24, LPK 50-20-25 and LPK 42-18-26 flowered significantly later than RP5973-20-9-8-24-12-7 (MTU1010 NIL).

However, all the breeding lines flowered significantly earlier compared to the male parent, RP6132. Similarly, the mean PH of the female parent (RP5973-20-9-8-24-12-7) and male parent (RP6132) were 78.7 cm and 66 cm, respectively. With respect to the improved breeding lines (F_5_s), PH varied from 96 cm (LPK 4-20-4; LPK 50-14-23) to 106 cm (LPK 50-20-25). Four breeding lines-LPK 3-19-3, LPK 4-20-4, LPK 38-5-18 and LPK 50-20-25 were shorter as compared to the female parent, and two breeding lines-LPK 50-14-23 and LPK 54-11-24 were similar to the female parent (RP5973-20-9-8-24-12-7) in terms of PH. Nine breeding lines-LPK 10-1-5, LPK 28-13-16, LPK 30-18-16, LPK 45-17-19, LPK 49-21-20, LPK 49-1-21, LPK 28-19-15, LPK 49-15-22, and LPK 42-18-26 were taller than the MTU1010 NIL, and all the breeding lines were taller as compared to the male parent (RP6132). The mean productive tiller numbers of the female parent (RP5973-20-9-8-24-12-7) and male parent (RP6132) were seven and four, respectively. Among the improved breeding lines, NPT varied from four (LPK 49-15-22; LPK 4-20-4) to nine (LPK 28-19-15; LPK 50-14-23). Four breeding lines-LPK 4-20-4, LPK 30-18-16, LPK 49-15-22, and LPK 42-18-26 possessed lesser tiller numbers than the MTU1010 NIL (RP5973-20-9-8-24-12-7). Interestingly, eight breeding lines-LPK 10-1-5, LPK 28-13-16, LPK 38-5-18, LPK 45-17-19, LPK 49-21-20, LPK 49-1-21, LPK 54-11-24, and LPK 50-20-25 showed significantly more productive tiller numbers per plant, as compared to the female parent.

Moreover, all the breeding lines had significantly higher number of productive tillers per plant compared to RP6132. The mean panicle length of RP5973-20-9-8-24-12-7 was 18 cm and 11.9 cm for RP6132. With respect to the improved breeding lines (F_5_s), PL varied from 13.3 cm (LPK 49-21-20) to 19.5 cm (LPK 30-18-16). Three breeding lines (LPK 4-20-4, LPK 30-18-16 and LPK 49-15-22) exhibited significantly higher values as compared to the MTU1010 NIL. One breeding line (LPK 49-1-21) showed panicle length similar to MTU1010 NIL.

All the breeding lines were significantly higher in terms of PL, compared to RP6132. The mean grain number per panicle of RP5973-20-9-8-24-12-7 and RP6132 were 112 and 86, respectively. In terms of the improved breeding lines, GPP varied from 70 (LPK 38-5-18) to 121 (LPK 30-18-16; 49-15-22). Two breeding lines (LPK 30-18-16 and LPK 49-15-22) exhibited significantly higher values as compared to the MTU1010 NIL, while the remaining lines were either equivalent to or lesser than MTU1010 NIL. The mean shoot weight of RP5973-20-9-8-24-12-7 and RP6132 were 16 g and 19 g, respectively. The mean DSW of the improved breeding lines (F_5_s) in low soil P plot varied from 14.7 g (LPK 54-11-24) to 23.3 g (LPK 10-1-5). A total of 10 breeding lines (LPK 50-20-25, LPK 3-19-3, LPK 4-20-4, LPK 28-19-15, LPK 28-13-16, LPK 30-18-16, LPK 45-17-19, LPK 49-21-20, LPK 49-1-21, and LPK 10-1-5) exhibited significantly higher values in terms of DSW, compared to MTU1010 NIL. Six breeding lines (LPK 50-20-25, LPK 4-20-4, LPK 10-1-5, LPK 30-18-16, LPK 49-21-20, and LPK 49-15-22) were significantly higher in terms of DSW as compared to RP6132. The mean DRW of RP5973-20-9-8-24-12-7 and RP6132 were 6.5 g and 1.7 g, respectively. In terms of improved breeding lines (F_5_s), DRW values varied from 3.3 g (LPK 30-18-16) to 6.8g (LPK 50-14-23).

One breeding line (LPK 50-14-23) exhibited significantly higher values than MTU1010 NIL. The mean RSR of the female parent and male parent were 0.41% and 0.09%, respectively. The improved breeding lines (F_5_s) showed RSR values ranging from 0.20% (LPK 30-18-16) to 0.45% (LPK 50-14-23). Interestingly, all the breeding lines exhibited significantly higher values as compared to RP6132 (which is devoid of *Pup1*) in terms of RSR (S 3A and 3B). The mean root volume (RV) of RP5973-20-9-8-24-12-7 (i.e., MTU1010 NIL possessing *Pup1*) and RP6132 were 46.3 ml and 28.7 ml, respectively. The mean RV of the improved breeding lines (F_5_s) varied from 34.3 ml (LPK 28-13-16) to 52 ml (LPK 49-1-21). Three breeding lines (LPK 4-20-4, LPK 30-18-16, LPK 49-1-21, LPK 49-15-22) were significantly higher, and one breeding line (LPK 42-18-26) was equivalent to the MTU1010 NIL (which possesses *Pup1*), in terms of RV. All the breeding lines were found to be significantly higher in terms of RV as compared to RP6132.

**Fig 4:**
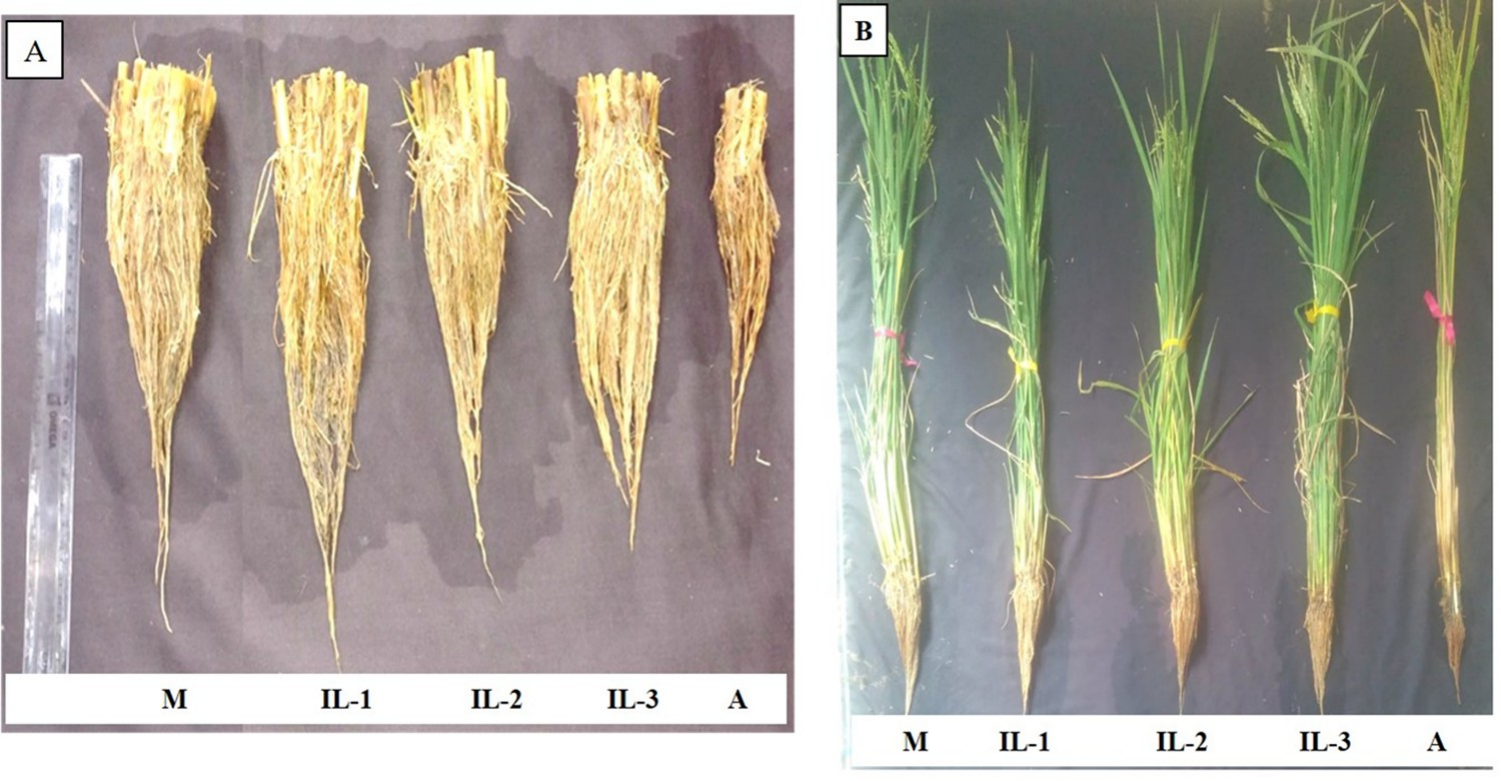
Performance of improved breeding lines under low P conditions. **(A)** Root morphology of Improved breeding lines (IL-1 to IL-3; at F_5_ generation) possessing *Pup1*, *Xa21* and *Pi54* under low soil P condition. **(B)** Improved breeding lines (IL-1 to IL-3; at F_5_ generation) possessing *Pup1*, *Xa21* and *Pi54* under low soil P condition), M- Tolerant check—MTU 1010 (RP5973-20-9-8-24-12-7; Pup1), A- Sensitive check—Akshayadhan (RP6132; *Xa21* & *Pi54*).

In the low soil P plot, the mean single plant yield (GY) of RP5973-20-9-8-24-12-7(i.e., MTU1010 NIL possessing *Pup1*) and RP6132 were 8.9g and 4.2g, respectively. With respect to the improved breeding lines (F5s) (possessing *Pup1*, *Xa21* and *Pi54*), the GY values varied from 4.4g (LPK28-19-15) to 9.9g (LPK49-1-21). Three breeding lines (LPK30-18-16, LPK49-1-21 and LPK49-15-22) were observed to be significantly higher in terms of single plant yield, compared to the MTU1010 NIL. All the breeding lines were higher compared to RP6132 (which is devoid of *Pup1*), in terms of yield per plant in the low soil P plot (Fig 4).

### Evaluation of improved breeding lines for agro-morphological traits

Under normal soil P condition, 15 breeding lines (possessing *Pup1*, *Xa21* and *Pi54*) derived from the cross of RP5973-20-9-8-24-12-7 x RP6132 were evaluated for 13 agro-morphological traits associated with low soil P tolerance at F_5_ generation. The ANOVA test revealed significant differences between the tolerant and sensitive parents and also the breeding lines for all the traits (Table 2).

Under normal soil P condition, the DFF of RP5973-20-9-8-24-12-7 (i.e., MTU1010 NIL possessing *Pup1*) and RP6132 (possessing *Xa21* and *Pi54*) were 106 and 121 days, respectively. With respect to the improved breeding lines (i.e., F5 generation lines possessing *Pup1*, *Xa21* and *Pi54*), the DFF varied from 105 days (LPK 30-18-16) to 125 days (LPK28-19-15). The breeding line LPK30-18-16 (105 days) flowered significantly earlier as compared to the female parent, while the 14 other breeding lines (LPK3-19-3, LPK4-20-4, LPK10-1-5, LPK28-19-15, LPK28-13-16, LPK38-5-18, LPK45-17-19, LPK49-21-20, LPK49-1-21, LPK49-15-22, LPK50-14-23, LPK54-11-24, LPK50-20-25, and LPK42-18-26) flowered significantly later, compared to RP5973-20-9-8-24-12-7 (MTU1010NIL). Ten breeding lines (LPK4-20-4, LPK10-1-5, LPK28-13-16, LPK30-18-16, LPK38-5-18, LPK49-1-21, LPK49-15-22, LPK54-11-24, LPK50-20-25, and LPK42-18-26) flowered significantly earlier, while five breeding lines (LPK3-19-3, LPK28-19-15, LPK45-17-19, LPK49-21-20, and LPK50-14-23) flowered significantly later, as compared to RP6132.

Similarly, the mean PH of RP5973-20-9-8-24-12-7 and RP 6132 were 99cm and 105cm, respectively. With respect to the improved breeding lines (F5s), PH varied from 96cm (LPK4-20-4, LPK 50-14-23) to 106 cm (LPK 50-20-25). Nine breeding lines (LPK 4-20-4, LPK 10-1-5, LPK 28-13-16, LPK 30-18-16, LPK 38-5-18, LPK 45-17-19, LPK 49-21-20, LPK 49-1-21, and LPK 50-14-23) were significantly taller than the MTU1010 NIL. Six breeding lines (LPK 3-19-3, LPK 28-19-15, LPK 49-15-22, LPK 54-11-24, LPK 50-20-25, and LPK 42-18-26) were shorter as compared to the female parent, while nine breeding lines (LPK 4-20-4, LPK 10-1-5, LPK 28-13-16, LPK 30-18-16, LPK 38-5-18, LPK 45-17-19, LPK 49-21-20, LPK 49-1-21, and LPK 50-14-23) were taller than the MTU1010 NIL, and all the breeding lines were shorter as compared to RP6132.The mean productive tiller numbers of RP5973-20-9-8-24-12-7 and RP6132 were 13 and 11, respectively. Among the improved breeding lines, the NPT varied from 10 (LPK 45-17-19, LPK 50-14-23, LPK 50-20-25) to 16 (LPK 49-15-22).

Ten breeding lines (LPK 3-19-3, LPK 10-1-5, LPK 28-19-15, LPK 28-13-16, LPK 45-17-19, LPK 49-21-20, LPK 49-1-21, LPK 50-14-23, LPK 54-11-24, and LPK 50-20-25) were either equivalent or lesser in terms of NPT as compared to MTU1010 NIL. Interestingly, 10 breeding lines (LPK 3-19-3, LPK 4-20-4, LPK 28-19-15, LPK 30-18-16, LPK 38-5-18, LPK 49-21-20, LPK 49-1-21, LPK 49-15-22, LPK 54-11-24, and LPK 42-18-26) showed significantly greater productive tiller number per plant as compared to RP6132. Five breeding lines (LPK 10-1-5, LPK 28-13-16, LPK 45-17-19, LPK 50-14-23, and LPK 50-20-25) had lesser productive tiller number per plant as compared to RP6132.The mean PL of RP5973-20-9-8-24-12-7 was 24 cm, and for RP6132, it was 21 cm. With respect to the improved breeding lines (F_5_s), PL varied from 20.67 (LPK 49-1-21) to 24.67 (LPK 4-20-4; LPK 42-18-26). One breeding line showed panicle length similar to MTU1010 NIL i.e., LPK 49-15-22. A total of 12 breeding lines (LPK 3-19-3, LPK 4-20-4, LPK 28-19-15, LPK 28-13-16, LPK 30-18-16, LPK 38-5-18, LPK 45-17-19, LPK 49-1-21, LPK 49-15-22, LPK 50-14-23, LPK 54-11-24, and LPK 42-18-26) were significantly higher in terms of PL, compared to RP6132. Three breeding lines (LPK 10-1-5, LPK 49-1-21, and LPK 50-20-25) showed significantly lesser PL, compared to the male parent. The mean grain number per panicle (NGP) of RP5973-20-9-8-24-12-7 and RP6132 were 221 and 174, respectively.

With respect to the improved breeding lines, GPP varied from 193 (LPK45-17-19) to 233 (LPK42-18-26). Four breeding lines (LPK4-20-4, LPK 30-18-16, LPK 49-15-22, and LPK 42-18-26) exhibited significantly higher values as compared to MTU1010 NIL, while the remaining 11 breeding lines displayed equivalent or lesser values than the MTU1010 NIL, with respect to NGP. All the breeding lines were significantly higher compared to the male parent in terms of GPP. The mean shoot weight of the female parent and male parent were 42g and 30.3g, respectively. The mean DSW of the improved breeding lines (F_5_s) in normal soil P plot varied from 30.7g (LPK50-14-23) to 49.3 g (LPK45-17-19). Five breeding lines (LPK28-19-15, LPK28-13-16, LPK45-17-19, LPK49-1-21, and LPK50-20-25) exhibited significantly higher values in terms of DSW as compared to MTU1010 NIL. A total of 11 breeding lines (LPK3-19-3, LPK4-20-4, LPK10-1-5, LPK28-19-15, LPK28-13-16, LPK30-18-16, LPK45-17-19, LPK49-21-20, LPK49-15-22, LPK50-20-25, and LPK42-18-26) were significantly higher in terms of DSW, compared to RP6132. Four breeding lines (LPK38-5-18, LPK49-1-21, LPK50-14-23, and LPK54-11-24) were observed to be significantly lesser in terms of DSW as compared to RP6132.The mean DRW of RP5973-20-9-8-24-12-7 and RP6132 were 8.2g and 7.6g, respectively.

With respect to the improved breeding lines (F_5_s), DRW values varied from 6.2g (LPK10-1-5) to 9.6g (LPK28-19-15, LPK28-13-16, and LPK49-1-21). Eleven breeding lines (LPK3-19-3, LPK4-20-4, LPK28-19-15, LPK28-13-16, LPK30-18-16, LPK45-17-19, LPK49-21-20, LPK49-1-21, LPK49-15-22, LPK54-11-24, and LPK42-18-26) exhibited significantly higher values than the male parent RP6132, with respect to DRW. The mean RV of RP5973-20-9-8-24-12-7 (i.e., MTU1010 NILpossessing*Pup1*) and RP6132 were 103 ml and 106.7 ml, respectively.

The mean RV of the improved breeding lines (F_*5*_s) varied from 80.3 ml (LPK50-20-25) to 110ml (LPK4-20-4). Two breeding lines (LPK3-19-3 and LPK4-20-4) were significantly higher in terms of RV than the MTU1010 NIL. One breeding line (LPK4-20-4) was significantly higher than male parent, while the other 14 breeding lines (LPK3-19-3, LPK10-1-5, LPK28-19-15, LPK28-13-16, LPK30-18-16, LPK38-5-18, LPK45-17-19, LPK49-21-20, LPK49-1-21, LPK49-15-22, LPK50-14-23, LPK54-11-24, LPK50-20-25, and LPK42-18-26) were significantly higher in terms of RV as compared to RP6132. In the normal plot, the mean single plant yield (GY) of RP5973-20-9-8-24-12-7 (i.e., NIL of MTU1010 possessing *Pup1*) and RP6132 were 23g and 20.3g, respectively.

**Fig 5:**
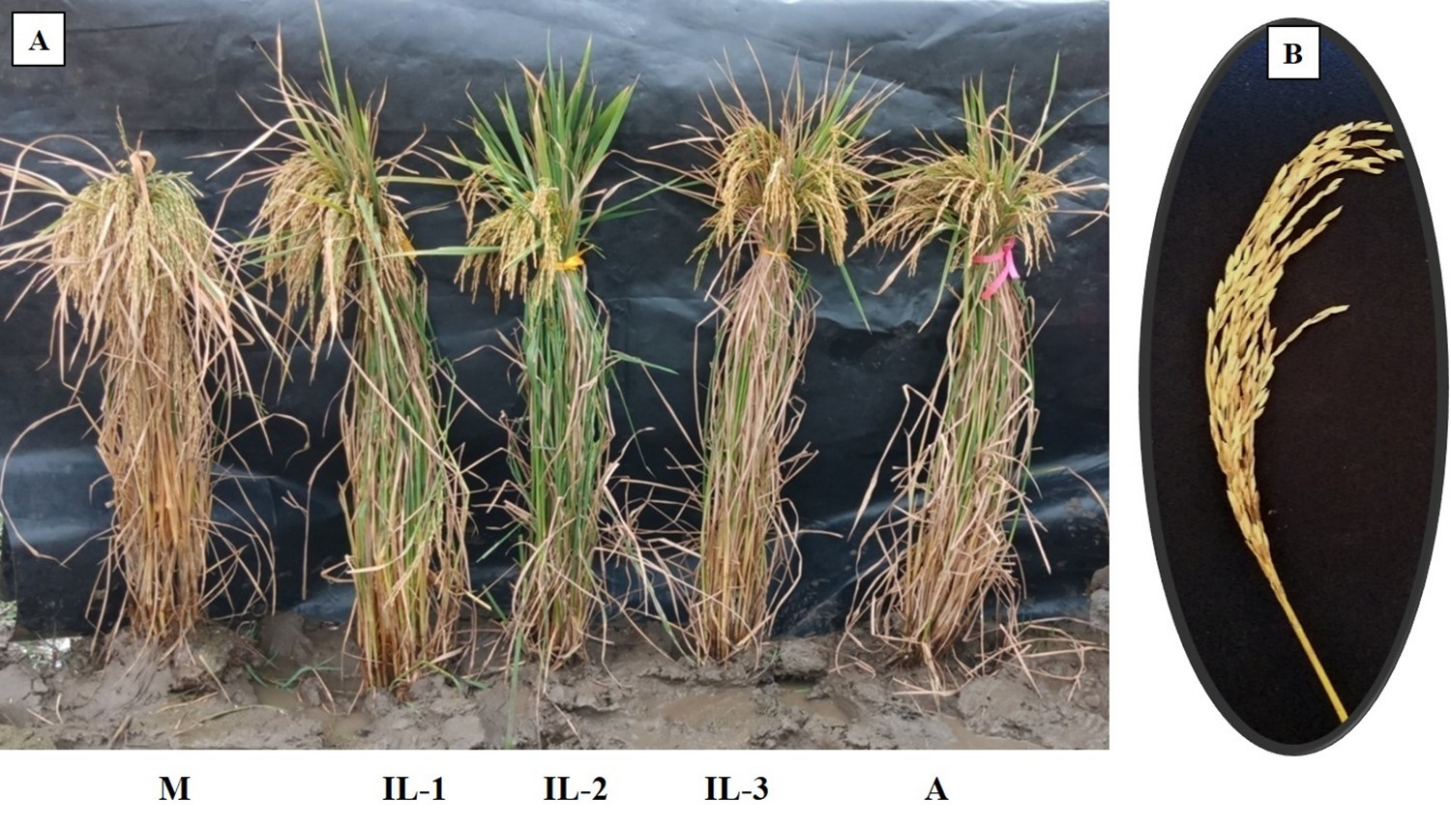
Improved breeding lines performing better than the recurrent parent. (A) Plant pictures (IL-1 to IL-3) and (B) Panicle picture of Improved breeding lines possessing *Pup1*, *Xa21* and *Pi54* at F_5_ generation. M- Female parent (MTU 1010 (Pup1) (RP5973-20-9-8-24-12-7), A- Male parent (Akshayadhan (*Xa21* & *Pi54*) (RP6132).

With respect to the improved breeding lines (F_5_ generation, possessing *Pup1*, *Xa21*and *Pi54*), the GY values varied from17.3g (LPK50-14-23) to 27.2g (LPK4-20-4; LPK42-18-26). Four breeding lines (LPK4-20-4, LPK30-18-16, LPK49-15-22, and LPK42-18-26) exhibited significantly higher values with respect to GY, and three breeding lines (LPK28-13-16, LPK45-17-19, and LPK54-11-24) were observed to be on par with MTU1010NIL. All the breeding lines were significantly higher compared to RP6132 (which is devoid of *Pup1*), with respect to single plant yield (Fig 5).

## Discussion

MTU1010 (also known as Cottondora Sannalu) is a mega-rice variety of India, released by APRRS, ANGRAU, Maruteru, Andhra Pradesh, India. It is of medium-duration maturity (120 days), has good grain quality (i.e., long slender grain type) and is suitable for planting in both wet and dry seasons, with a high yielding capacity of six to seven tons/ha. The variety is estimated to be cultivated in >12% of Indian rice areas, due to its wider adaptability along with good level of tolerance to BPH [17]. However, the variety is highly susceptible to BB and has only moderate level of tolerance to blast disease, both of which cause significant yield losses in many states of India. Further, MTU1010 is also highly sensitive to low soil P conditions [18]. We had earlier developed a NIL of MTU1010 (RP5973-20-9-8-24-12-7) through the targeted transfer of the low soil P tolerance gene *Pup1* [18] and NIL of Akshayadhan (RP6132; slender grain type rice, similar to MTU1010 in terms of grain quality and yield), having resistance against BB (conferred by *Xa21*) and blast (conferred by *Pi54*) [8].

Using these two lines as parents, we improved MTU1010 for tolerance to low soil P conditions along with improvement of resistance against BB and blast, in the present study. In the present-day scenario of emergence of BB and blast as major production constraints in rice, coupled with increasing cost of P fertilizers, along with low availability of soil P in many rice growing areas, development of multiple stress resistant/tolerant varieties/breeding lines has assumed significance in rice breeding. Despite significant increase in production and productivity of different rice varieties, over the last five decades, in recent years, the yield and productivity levels of Indian rice varieties have stagnated. One of the main reasons for this is the susceptibility of most of the popularly cultivated mega-rice varieties, like Samba Mahsuri, Swarna, MTU1010, etc., to pests and diseases and various abiotic stresses [7], such as low soil P levels. In order to sustain the yield levels of these elite and popular rice varieties, it is imperative to develop disease resistant and low P tolerant versions. Resistance breeding is considered the most economical and eco-friendly strategy for management of biotic stresses [19].

While resistance breeding is the only option for management of BB [14], in case of blast disease, even though many effective fungicides are available, they are often costly and cannot be afforded by small and medium farmers. In such a scenario, selection of resistance genes/gene combinations for deployment is important to sustain rice cultivation in areas prone to blast and BB diseases. With this objective, in the present study, breeding lines possessing resistance to blast and BB diseases, along with tolerance to low soil P, and having yield and grain quality similar to or better than MTU1010, have been developed through the strategy of MAPB, coupled with phenotype-based selection for agro-morphological traits.

*Pi54* is a major blast resistance gene derived from a Vietnamese rice line, Tetep. The gene is known to be very effective against the blast pathogen isolates prevalent in India [20, 21]. Along with *Pi54*, we also considered deployment of another major dominant BB resistance gene, *Xa21*, which confers broad-spectrum resistance against Indian isolates of *Xoo* [22, 23]. *Xa21* was originally introgressed from an accession of African wild rice, *Oryza longistaminata*. In addition to *Pi54* and *Xa21*, a major QTL/gene associated with tolerance to low soil P, *Pup1* (*Phosphorus uptake1*), was also deployed in this study for enhancing the performance in low soil P conditions. *Pup1* was identified on 12th chromosome of rice of the *Aus* variety, Kasalath, fine-mapped, cloned, and characterized [24–26]. The QTL/gene was introgressed through marker-assisted breeding into the *Indica* rice varieties, IR64, IR72, Dodokan, Batur, and Situ [9]. Phenotypic evaluation of the introgressed lines suggested that *Pup1* is effective in different genetic backgrounds, in both irrigated and rain-fed environments. Hence, in this study, even though MTU1010 is a variety released for irrigated ecosystem, we deployed *Pup1* in the variety, so that the breeding lines could perform well in soils with low available P.

We had earlier developed improved versions of elite rice varieties and hybrid rice parental lines, which showed excellent resistance against BB and blast [21–23]. We selected one major gene each conferring resistance against BB (i.e., *Xa21*) and blast (i.e., *Pi54*), as there are no reports of large-scale break down of resistance conferred by these resistance genes in India [24, 25].

We had earlier transferred *Pup1* QTL into the genetic background of elite rice varieties [7]. To date, *Pup1* is the only major QTL/gene available for improvement of low soil P tolerance. Based on the above-mentioned points, the present study was formulated in order to combine tolerance to low soil P and resistance against BB and blast into the genetic background of NIL of MTU1010, by crossing RP5973-20-9-8-24-12-7 x RP6132 through MAPB (i.e., for *Pup1*, *Xa21*, and *Pi54*), coupled with phenotypic selection for recovery of superior traits present in MTU1010 (i.e., for high yield, good plant type and a desirable long-slender grain type).

Analysis of variance among the F_5_ lines revealed significant differences between the parents and breeding lines for days to fifty percent flowering, plant height, number of productive tillers, panicle length, root length, dry root weight, thousand grain weight and grain yield per plant under low soil P condition, but significant differences were not observed for dry shoot weight. Notably, most of the improved breeding lines (possessing *Pup1*, *Xa21*, and *Pi54*) in normal soil P and low soil P plots were found on par, and few lines were found to be superior with respect to yield, as compared to MTU1010 NI L (Tables 2 and 3).

**Table 3:** Evaluation of the improved breeding lines (F_5_ generation) for key agro-morphological traits in plot with normal soil P levels at ICAR-IIRR during wet season 2018.

Genotype/ Entry	DFF	PH	NPT	PL	RV	RL	DSW	DRW	RSR	NGP	TGW	SPY	GT
RP5973-20-9-8-24-12-7	106.0 ± 0.6	99.2 ± 0.2	13 ± 0.3	24.0 ± 0.2	103.0 ± 0.6	22.2 ± 0.6	43.2 ± 0.4	8.2 ± 0.3	0.16± 0.0	221 ± 0.6	22.3 ± 0.3	23.0 ± 0.3	LS
RP6132	121.0 ± 0.6	105.3±0.3	12 ± 0.3	22.0 ± 0.6	106.7 ± 0.7	30.0 ± 0.6	31.6 ± 0.9	7.6 ± 0.2	0.12± 0.0	174 ± 0.9	20.7 ± 0.3	20.3 ± 0.9	LS
ISM	116.0 ± 0.9	78.8 ±0.9	9 ± 0.6	19.9 ± 0.5	42.8 ± 0.3	23.5 ± 0.6	37.0 ± 0.4	4.0 ± 0.6	0.11± 0.0	205 ± 0.6	14.4 ± 0.3	20.7 ± 0.2	MS
SWARNA	125.0 ± 0.9	77.2 ±0.6	10 ± 0.6	20.4 ± 0.3	68.5 ± 0.3	23.7 ± 0.3	48.9 ± 0.1	5.6 ± 0.1	0.12± 0.0	220 ± 0.6	20.2 ± 0.3	23.4 ± 0.6	SB
LPK 3-19-3	124.0 ± 0.6	104.3±0.3	12 ± 0.3	23.0 ± 0.6	105.7 ± 0.3	22.5 ± 0.3	41.9 ± 0.4	9.5 ± 0.3	0.19± 0.0	204 ± 0.6	21.8 ± 0.4	21.0 ± 0.6	LS
LPK 4-20-4	106.0 ± 0.6	102.3±0.3	16 ± 0.3	24.0 ± 0.6	98.0 ± 0.6	24.0 ± 0.6	32.9 ± 0.5	8.6 ± 0.2	0.22± 0.0	213 ± 0.9	22.5 ± 0.6	23.7 ± 0.4	LS
LPK 10-1-5	110.3± 0.3	98.7±0.3	10 ± 0.6	21.3 ± 0.3	85.3 ± 0.3	21.8 ± 0.4	36.0 ± 0.6	6.2 ± 0.4	0.17± 0.0	206 ± 0.7	21.5 ± 0.3	18.0 ± 0.6	LS
LPK 28-19-15	125.7± 0.3	100.0±0.6	12 ± 0.3	22.7 ± 0.3	90.3 ± 0.9	20.0 ± 0.3	48.4 ± 0.8	9.6 ± 0.3	0.17± 0.0	211 ± 0.6	21.5 ± 0.3	21.0 ± 0.5	LS
LPK 28-13-16	111.7± 0.3	97.0±0.6	11 ± 0.6	22.0 ± 0.6	82.3 ± 0.9	22.3 ± 0.7	49.0 ± 0.3	9.6 ± 0.3	0.17± 0.0	220 ± 0.9	19.0 ± 0.6	23.0 ± 0.5	LS
LPK 30-18-16	105.0± 0.6	98.3±0.3	14 ± 0.9	27.3 ± 0.3	102.0 ± 0.6	24.0 ± 0.6	38.4 ± 0.5	8.3 ± 0.3	0.18± 0.0	224 ± 0.9	22.6 ± 0.4	27.0 ± 0.5	LS
LPK 38-5-18	111.7 ± 0.9	96.7±0.7	13 ± 0.6	23.7 ± 0.3	86.0 ± 0.6	19.0 ± 0.6	31.3 ± 0.2	7.3 ± 0.4	0.20± 0.0	209 ± 0.9	22.0 ± 0.3	22.3 ± 0.2	LS
LPK 45-17-19	125.0 ± 0.6	98.3±0.3	10 ± 0.3	22.3 ± 0.9	83.0 ± 0.6	20.3 ± 0.3	49.3 ± 0.9	9.4 ± 0.3	0.15± 0.0	193 ± 0.6	21.7 ± 0.3	23.0 ± 0.6	LS
LPK 49-21-20	121.3± 0.9	97.0±0.6	11 ± 0.7	21.7 ± 0.7	85.7 ± 0.3	22.5 ± 0.6	36.2 ± 0.4	8.3 ± 0.4	0.16± 0.0	213 ± 0.9	20.7 ± 0.9	20.7 ± 0.9	LS
LPK 49-1-21	111.3± 0.9	102.3±0.3	15 ± 0.6	24.7 ± 0.3	102.0 ± 0.6	23.5 ± 0.5	47.0 ± 0.6	9.5 ± 0.3	0.26± 0.0	233 ± 0.6	22.5 ± 0.3	27.2 ± 0.4	LS
LPK 49-15-22	111.7 ± 0.9	96.0±0.6	15 ± 0.6	24.7 ± 0.3	110.0 ± 0.6	23.3 ± 0.4	31.3 ± 0.3	8.0 ± 0.6	0.19± 0.0	228 ± 0.6	22.5 ± 0.3	27.2 ± 0.8	LS
LPK 50-14-23	123.0± 0.6	96.0±0.6	10 ± 0.3	23.0 ± 0.6	82.7 ± 0.9	22.7 ± 0.3	30.7 ± 0.3	7.0 ± 0.1	0.14± 0.0	211 ± 0.3	21.9 ± 0.1	17.3 ± 0.7	LS
LPK 54-11-24	112.7 ± 0.3	100.3±0.7	11 ± 0.6	23.7 ± 0.3	91.7 ± 0.3	22.8 ± 0.6	31.3 ± 0.3	8.2 ± 0.6	0.20± 0.0	196 ± 0.9	18.8 ± 0.6	23.0 ± 0.6	LS
LPK 50-20-25	115.0± 0.6	106.0±0.6	10 ± 0.3	21.0 ± 0.6	80.3 ± 0.3	19.3 ± 0.6	47.7 ± 0.3	7.0 ± 0.6	0.16± 0.0	216 ± 0.6	21.7 ± 0.7	22.8 ± 0.4	LS
LPK 42-18-26	117.7± 0.9	97.3±0.9	12 ± 0.6	20.7 ± 0.3	82.3 ± 0.7	22.7 ± 0.3	31.7 ± 0.3	9.6 ± 0.3	0.18± 0.0	218 ± 0.3	22.3 ± 0.3	17.6 ± 0.7	LS
**CV%**	1.00	0.92	7.48	3.66	1.15	3.84	2.23	8.02	9.18	0.50	3.43	4.51	
**LSD at 5%**	1.926	1.48	1.48	1.37	1.69	1.44	1.46	1.00	0.02	1.73	1.19	1.66	
**F value**	108.43	212.04	13.42	14.09	713.29	21.27	193.51	16.93	17.77	526.88	21.51	25.86	
**p value (1%)**	< .0001	< .0001	< .0001	< .0001	< .0001	< .0001	< .0001	< .0001	< .0001	< .0001	< .0001	< .0001	

RP5973-20-9-8-24-12-7 Female parent; RP (6132)- Male Parent; DFF-days to 50% flowering, PH-plant height (cm), NPT-number of productive tillers per plant, PL-panicle length (cm), RV-root volume (ml), DSW-dry shoot weight (gr), DRW-dry root weight (gr), RSR-root to shoot ratio (%), NGP-number of grains per panicle, TGW-1000 grain weight, SPY-single plant yield, LS-Long slender, MS-Medium slender; CV-Coefficient of variance; CD-Critical differential at 5% probability level. ±Standard error and values given are mean of three replications.

Four breeding lines (LPK4-20-4, LPK30-18-16, LPK49-15-22, and LPK42-18-26) showed significantly higher single plant yield as compared to MTU1010NIL in normal soil Pcondition, while three breeding lines (LPK30-18-16, LPK49-1-21, LPK49-15-22) showed significantly higher single plant yield in low soil P condition. All the breeding lines were observed to be higher in terms of single plant yield as compared to the male parent RP613. In most of the improved breeding lines (i.e., F_5_ lines), there was no apparent yield penalty associated with the presence of BB (*Xa21*) and blast (*Pi54*) resistance genes, along with low P (*Pup1*) tolerance.

Two breeding lines (LPK30-18-16 and LPK49-15-22) (possessing *Xa21*, *Pi54*, and *Pup1*) in F_5_ generation displayed high yield under both low soil P and normal soil P conditions, possessed long slender grain type, and showed high level of resistance to blast and BB diseases. This indicates that stringent marker-assisted selection in the early generation coupled with stringent phenotypic selection in the later generations can result in identification of superior lines/segregants. The two lines mentioned above are expected to perform well in areas prone to the two diseases, and also in upland areas of rice cultivation, where there is low availability of soil phosphorous [26]-for instance, in the nascent state of Telangana-during both wet and dry seasons, due to their short duration, similar toMTU1010.

Further, the breeding lines of MTU1010 NIL possessing BB and blast resistance along with *Pup1* can also serve as good donors for targeted transfer of the major genes/QTL to other elite rice varieties cultivated in the Telangana state. The promising breeding lines developed in this study will be advanced through the pedigree method of breeding for possible multiplication and evaluation through multi-location trials in the state of Telangana, and also across the country through All India Coordinated Research Improvement Project (AICRIP). Further, these lines can also serve as excellent donor for resistance against BB, blast and tolerance to low soil P, as they possess, *Xa21*, *Pi54* and *Pup1*, respectively.

## Supporting information

S1 Data (XLSX)
